# Supplementary material for: The causal role of circulating inflammatory markers in osteoporosis: a bidirectional Mendelian randomized study
Source: Front Immunol. 2024 Jul 18;15:1412298. doi: 10.3389/fimmu.2024.1412298 (PMC11291241; doi:10.3389/fimmu.2024.1412298)
Supplement: Supplementary file 1 [file DataSheet_1.zip › supplementary material/CIM-OSTEOPOROSIS /GCST90274792.csv_leave_one_out.pdf]

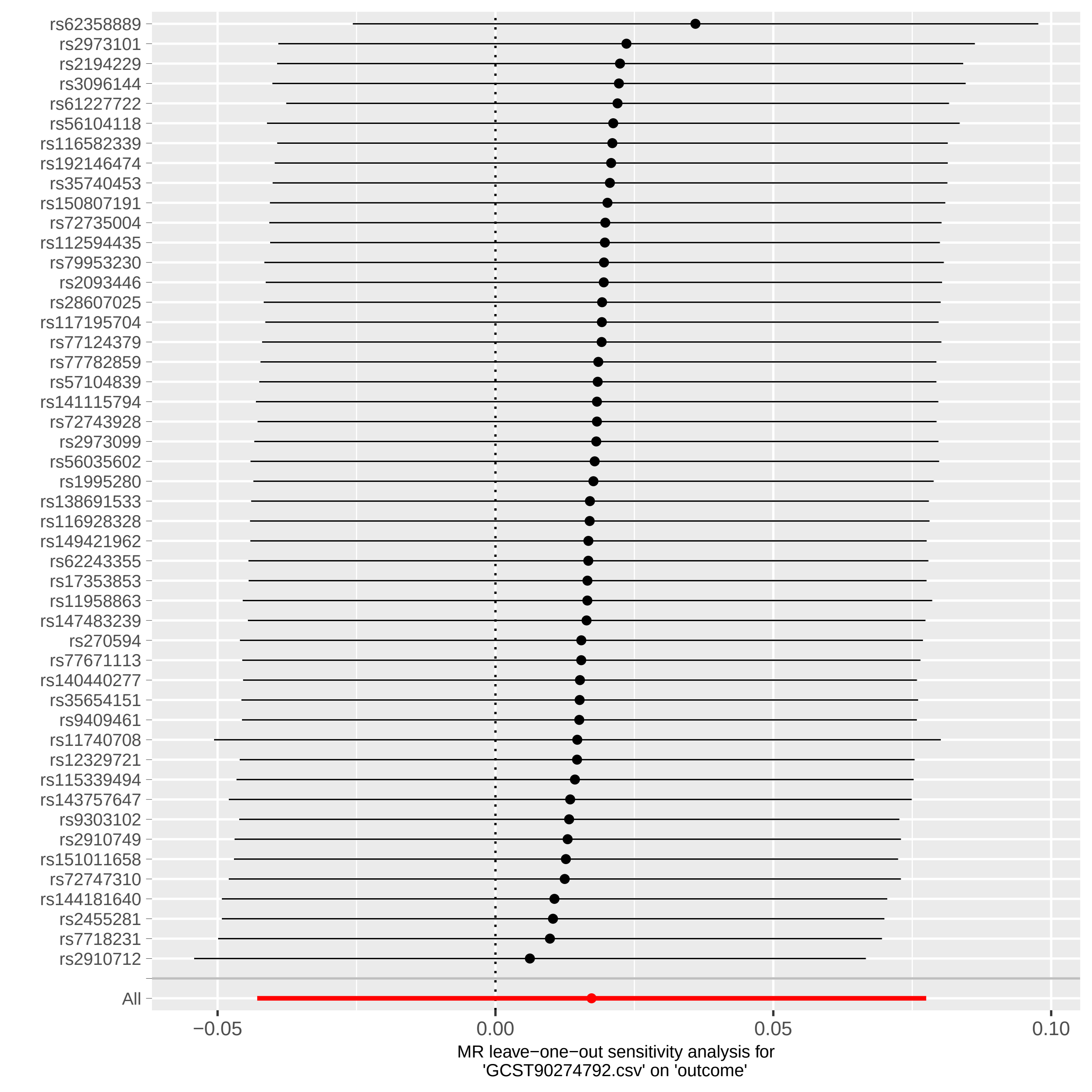
rs62358889
rs2973101
rs2194229
rs3096144
rs61227722
rs56104118
rs116582339
rs192146474
rs35740453
rs150807191
rs72735004
rs112594435
rs79953230
rs2093446
rs28607025
rs117195704
rs77124379
rs77782859
rs57104839
rs141115794
rs72743928
rs2973099
rs56035602
rs1995280
rs138691533
rs116928328
rs149421962
rs62243355
rs17353853
rs11958863
rs147483239
rs270594
rs77671113
rs140440277
rs35654151
rs9409461
rs11740708
rs12329721
rs115339494
rs143757647
rs9303102
rs2910749
rs151011658
rs72747310
rs144181640
rs2455281
rs7718231
rs2910712
All
−0.05
0.00
0.05
0.10
MR leave−one−out sensitivity analysis for
'GCST90274792.csv' on 'outcome'
